# Identifying Space Use at Foraging Arena Scale within the Home Ranges of Large Herbivores

**DOI:** 10.1371/journal.pone.0128821

**Published:** 2015-06-11

**Authors:** Norman Owen-Smith, Jodie Martin

**Affiliations:** Centre for African Ecology, School of Animal, Plant and Environmental Sciences, University of the Witwatersrand, Wits, South Africa; Indian Institute of Science, INDIA

## Abstract

An intermediate spatiotemporal scale of food procurement by large herbivores is evident within annual or seasonal home ranges. It takes the form of settlement periods spanning several days or weeks during which foraging activity is confined to spatially discrete foraging arenas, separated by roaming interludes. Extended by areas occupied for other activities, these foraging arenas contribute towards generating the home range structure. We delineated and compared the foraging arenas exploited by two African large herbivores, sable antelope (a ruminant) and plains zebra (a non-ruminant), using GPS-derived movement data. We developed a novel approach to specifically delineate foraging arenas based on local change points in distance relative to adjoining clusters of locations, and compared its output with modifications of two published methods developed for home range estimation and residence time estimation respectively. We compared how these herbivore species responded to seasonal variation in food resources and how they differed in their spatial patterns of resource utilization. Sable antelope herds tended to concentrate their space use locally, while zebra herds moved more opportunistically over a wider set of foraging arenas. The amalgamated extent of the foraging arenas exploited by sable herds amounted to 12-30 km^2^, compared with 22-100 km^2^ for the zebra herds. Half-day displacement distances differed between settlement periods and roaming interludes, and zebra herds generally shifted further over 12h than sable herds. Foraging arenas of sable herds tended to be smaller than those of zebra, and were occupied for period twice as long, and hence exploited more intensively in days spent per unit area than the foraging arenas of zebra. For sable both the intensity of utilization of foraging arenas and proportion of days spent in foraging arenas relative to roaming interludes declined as food resources diminished seasonally, while zebra showed no seasonal variation in these metrics. Identifying patterns of space use at foraging arena scale helps reveal mechanisms generating the home range extent, and in turn the local population density. Thereby it helps forge links between behavioural ecology, movement ecology and population ecology.

## Introduction

Spatiotemporal patterns of food procurement may be identified across a hierarchy of scales, from food items consumed at feeding stations to annual or lifetime ranges [1–3]. Much attention has been focused on the delineation of home ranges, manifested over annual, seasonal or briefer periods [4–7]. Classically, a home range is defined as the area normally traversed by an animal during its routine activities [8], but what is “normal” or “routine” is left vague, as well as the time frame over which the home range is defined [9]. Home ranges are commonly delineated phenomenologically using kernel density isopleths without regard to the mechanisms generating local concentrations of use [10]. Locations where animals remain immobile contribute disproportionately to the utilization distribution, while other sections of the area encompassed may be occupied merely in transit. Meeting certain needs may require temporary excursions, e.g. to reach water for drinking [11]. In order to relate the extent of the area traversed to the needs fulfilled, places occupied must be associated with the activities performed and hence the benefits derived. Foraging commonly constitutes by far the greatest portion of mobile activity, especially among large mammalian herbivores [12], and hence shapes the basic features of space occupation. Progress has been made towards achieving a mechanistic understanding of the processes generating home ranges for mammalian carnivores foraging outwards from dens [13], but not yet for herbivores exploiting food resources that are localised in space and seasonally variable in their nutritional value.

Fine-scale foraging behaviour has been analysed invoking principles of evolutionary adaptation to explain the range of food types consumed, feeding durations within patches where food is concentrated, and search paths between such patches [14–15]. Intermediate between the feeding patch, exploited over durations of several minutes, and the home range, occupied for seasonal or longer periods, is a spatiotemporal scale identified by Bailey et al. [2] as the area covered during foraging spells enduring several hours. In the context of foraging theory these areas, which we will label *foraging arenas* (FAs), represent the bounded context for comparing locally diminishing rates of food gain with what might potentially be obtained elsewhere. Our use of this label is distinct from that of Ahrens et al [16], who emphasised spatial contrasts in security from predation in marine ecosystems. Over the course of a day, foraging activity alternates with spells of resting as well as other maintenance activities [12]. Upon resuming foraging, an animal may either remain longer in the FA where it had previously sought food, or relocate seeking a new FA. In this way, settlement periods within FAs become extended over several successive days, with allowance for interruptions by other activities [3,17]. The set of FAs exploited in this way is dependent on the spatial distribution and seasonal production of food resources and how their availability is affected by patterns of consumption. Concepts of marginal value [18] can potentially be applied to durations of patch use and return intervals to patches at FA scale if the times of entry and departure from such places can be defined objectively. The overall extent of the home range utilized depends on the number and size of the distinct FAs exploited, the spatial dispersion of these FAs, and additional places occupied to fulfil other needs.

Opportunities to analyse the movement patterns of animals in fine spatiotemporal detail over periods spanning a year or longer have been opened by innovations in Global Positioning System (GPS) telemetry [19]. The developing field of movement ecology is concerned with where animals should stay and how long they should remain in these places before relocating elsewhere [20]. Motivational states underlying movement patterns may be identified from displacement distances and turning angles between sequential locations [21–23]. Movements are expected to become slower and trajectories more tortuous in places providing abundant food, and faster and more directed during travel between these localities. This approach becomes less valid for multi-day periods during which animals have engaged in distinct activities during the course of a day, potentially conducted in different places at different times. Nevertheless, animals remain stationary at some spatiotemporal scale while they continue exploiting particular FAs.

Scaling issues in the identification of stationary activity were addressed in some detail by Benhamou [24]. Concepts of first-passage time, defined as the time required for an animal to cross a circle with a given radius from some initial point, have been used to identify spatial cohesion among sequences of locations [25]. This residence time approach was extended by Barraquand and Benhamou [26] and coupled with a statistical procedure developed by Lavielle [27] to identify breakpoints indicating when stationary periods started and ended. In a further development, Benhamou and Riotte-Lambert [28] adapted kernel density estimators of utilization distributions to distinguish places used intensely because of prolonged residence times from those re-visited frequently. Applications of these methods thus far have been to fine-scale movements revealed by GPS data recorded at hourly or sub-hourly intervals [29].

A drawback in applying the concept of first passage time is the need to specify the radius of the circle used *a priori*. FAs can potentially vary quite widely in their extent, dependent on animal species, habitat type and season. Settlement periods within FAs are visually evident in time traces of sequential locations from blocks showing no spatial drift with time, i.e., d(*x*,*y*)/d*t* ~ 0, where *x* and *y* define location in longitude and latitude, and time *t* encompasses some multi-day period. In order to delineate these stationary periods, their start and end points need to be identified bearing in mind the core question in movement ecology—did the animal stay where it had been, or move on and away?

In this paper, we consider the space use patterns at FA scale of two large grazers showing distinct movement tendencies: sable antelope (*Hippotragus niger*), which are rather sedentary ruminants, and plains zebra (*Equus quagga*), which are much more mobile non-ruminants. We compare the delineation of FAs obtained using a novel approach based on local change points in time in the spatial proximity of successive locations with that provided by two published methods using either residence times based on first passage [26] or local kernel density estimates [28]. Having thereby obtained durations of settlement and extents of the patches exploited at FA scale, we illustrate how informative measures of foraging performance can be derived, including (a) local intensities of exploitation in foraging time spent per unit area, and (b) proportion of time spent settled within FAs versus roaming. Because of the attenuation in food availability for large herbivores from the wet season through the dry season, we expected to find the following seasonal patterns shown by both herbivore species:

- Settlement durations would decrease as food availability within FAs diminished seasonally, until too little food remained elsewhere.
- The extent of the FAs exploited would expand as exploitation of food resources became widened seasonally.
- Exploitation intensities within FAs (i.e the ratio between settlement duration and size of FAs) would diminish correspondingly with the seasonal progression.
- FAs would be more fragmented with more relocation movements during the dry season than in the wet season, meaning that the proportion of days spent within FAs relative to roaming interludes between them would decrease as the dry season advanced.
- As non-ruminants with greater tolerance for low food quality, zebra would be less responsive to seasonal variation in food resources than ruminant sable.

## Materials and Methods

### The data

GPS collars transmitting location records through the mobile telephone (GSM) network (supplier: http://www.awt.co.za) were placed on one female sable antelope in each of seven distinct herds inhabiting three widely separated regions of the Kruger National Park in South Africa, and on one female zebra in each of six distinct herds in one of these regions. The initial collars were replaced on some of the sable to extend the GPS data coverage over more than one year (Table 1). Collars initially recorded GPS locations routinely at six-hourly intervals, while replacement collars provided hourly data. Because both of these ungulates form cohesive herds, the movements of the collared individuals represented the movements of the herds with which they were associated. Location records were sub-sampled to represent the times of day when the animals were most likely to be engaged in foraging activity, i.e. 08:00 and 20:00 hours, guided by the bimodal diel activity schedules shown by the two ungulate species [12]. Relocation movements tended to take place at dawn, shortly before the 08:00 locations, and towards dusk, preceding the 20:00 locations. Location error was usually not greater than 10 m [30], and fewer than 6% of scheduled location records were missing. Missing data were reduced to <3% using positions recorded one hour earlier or later than the standard times if available.

**Table 1:** Periods during which location data were supplied for the individual females representing distinct herds in the three study areas.

Species	Study area	GPS collar(s)	Herd size	Period spanned	Duration (months)
Sable	Central	Tal01	15	2004-11-26 to 2005-11-05	11.5
Sable	Central	Tal02	6	2004-11-26 to 2006-02-23	15
Sable	South	PK81/140/285	7–13	2005-11-14 to 2008-03-27	33.5
Sable	South	PK144	7	2006-05-24 to 2007-03-07	9.5
Sable	South	PK148/284	6–9	2006-05-23 to 2009-03-21	34
Sable	South	PK151/286	7–10	2006-05-23 to 2008-09-29	28
Sable	North	PM143	18–21	2006-05-22 to 2007-12-18	19
Zebra	North	PM141	6–7	2006-05-23 to 2007-09-29	16
Zebra	North	PM142	6–7	2006-05-23 to2008-11-06	30
Zebra	North	PM147	6–7	2006-05-22 to 2007-07-07	13.5
Zebra	North	PM277	6–7	2007-06-18 to 2010-10-14	40
Zebra	North	PM280	6–7	2007-06-18 to 2008-09-08	15

Our research did not entail animal observations. We drew on a database of GPS locations established by previous studies (http://dataknp.sanparks.org).

Rainfall patterns in Kruger Park defined a wet season extending from October or November through March or April, and a dry season extending from May into October. Accordingly, we grouped months into the wet season (December-March), early or cool dry season (April-July), and late or hot dry season (August-November). Typically less than 20% of the annual rainfall falls during the dry season months, so that grass growth ceases and remaining grass becomes progressively brown and dry. The total annual rainfall during the 2005/6 seasonal cycle (July-June) was about 25% above the long-term mean (1960–2007), and that through 2006/7 was 33% below the mean. The first wet season rain was delayed until early November in 2006, but occurred in late September in 2007. The seasonal cycle 2003/2004 spanned by the earliest sable collars placed was exceptionally dry.

### Home range estimation

The overall home ranges of the sable and zebra herds were assessed by the kernel density method [31] using the package adehabitat [32] for R [33]. Ninety percent isopleths were chosen for the kernel density estimation of the extent of these home ranges. Utilization distributions in three dimensions were plotted using Systat 11 software [34].

### Local change points in spatial location

**Fig 1:**
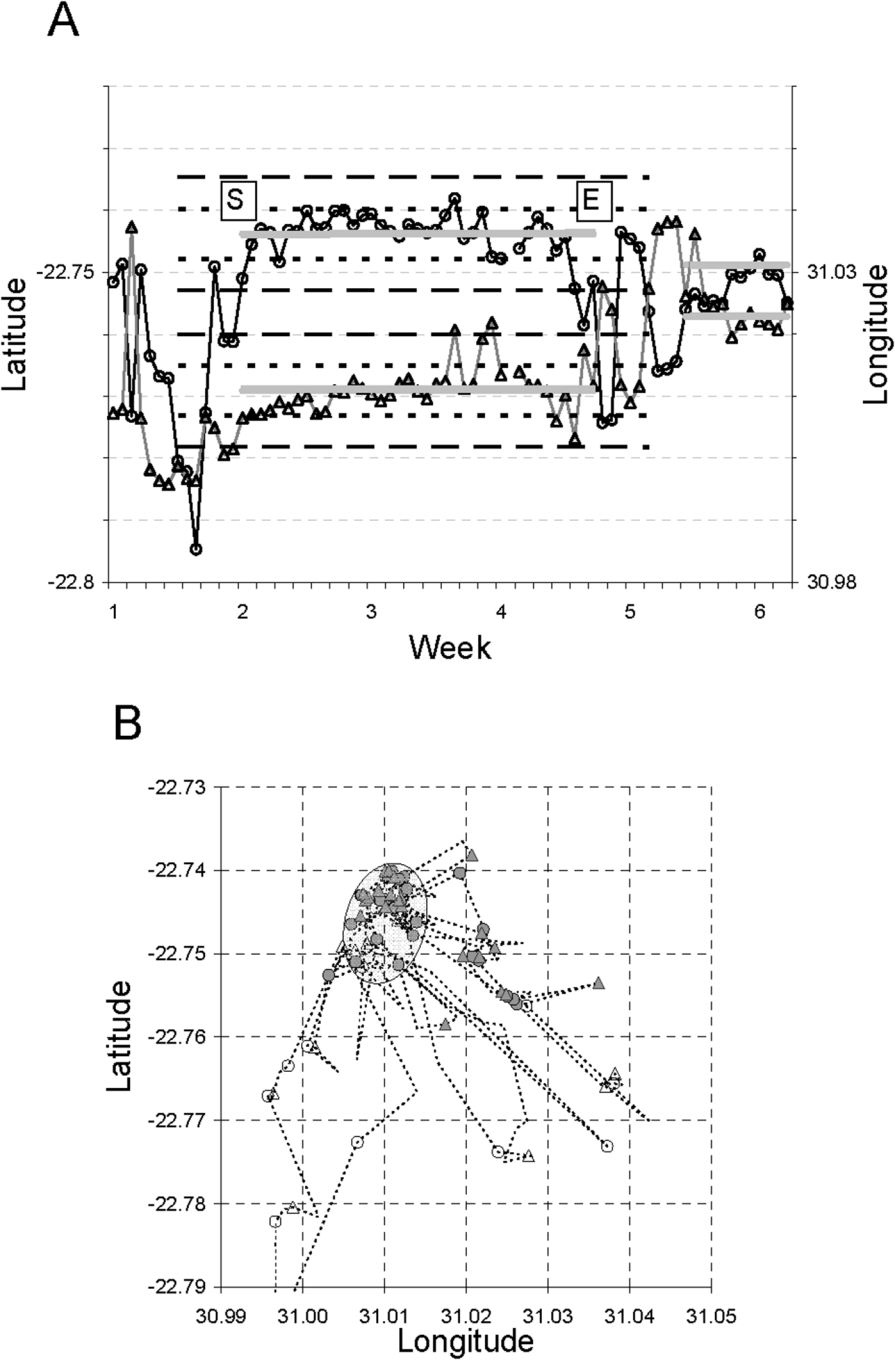
Identifying local change points to define settlement periods within foraging areas from sequential GPS locations. (a) Time traces for latitude (circles) and longitude (triangles) at two times of day (08:00 h and 20:00 h) for the sable antelope showing (i) foraging area centroids in latitude and longitude coordinates (broad grey lines), (ii) mean radial distances from the centroid during settlement (dotted lines) and (iii) settlement constraints represented by the mean radial distance plus two standard deviations (dashed lines). Labels S and E indicate the start and end of the settlement period. Note excursions slightly beyond the FA limits before final departure, as well as transient return during the roaming interlude before the next settlement period. (b) Mapped trajectory of GPS locations of the sable for the same period. Circles represent morning (08:00) locations and triangles evening (20:00) locations, while dotted lines connect intervening locations. Filled symbols indicate locations during the settlement period, including brief excursions. The foraging arena was entered from the south and departure was to the south-east. Ellipse encloses the foraging area extent of 1.2 km^2^.

To identify change points in spatial location, we used the distance of the current position relative to the distribution of preceding or succeeding positions within adjoining time windows. Specifically, the local reference points used were the centroids of sets of GPS positions (i.e. the arithmetic means of latitude and longitude) within these time windows. As a practical minimum, five-day windows were used to provide 10 position records (fewer if 1–2 were missing) and used initially for assessing the local distribution of distances from the centroid. Departure from the FA was established looking back over the preceding time window and initiation of settlement with the FA looking forward over the succeeding time window. If the radial distance of a focal position from the centroid was greater than the mean plus two standard deviations around this mean of the neighbouring locations, a local change point was flagged. Entries into and exits from FAs identified in this way were generally associated with abrupt contractions or surges in half-day (12-h) displacement distances. The application of this procedure is illustrated in Fig 1.

Constraint settings were used to ensure that the FAs identified conformed to the scale of the patches occupied by each collar-bearing animal. They excluded change points based on distances too short to be effectively departures, and joined neighbouring FAs too close to be distinct. The log-frequency distribution of half-day displacements was used initially for guidance, looking for a breakpoint in this distribution between within-patch and between-patch displacements. For the sable herd chosen for illustration, the modal half-day displacement was 0.2–0.3 km, and the break-point between displacement modes seemed to lie around 2 km (S1 Fig). The optimal setting was sought by exploring how the number of FAs distinguished changed following stepped adjustments to the constraint settings. If the setting was too fine, departures tended to be flagged prematurely, thereby attenuating some settlement durations below the minimum of five days. If the setting was too coarse, neighbouring FAs became joined, also reducing the number of settlement periods identified. The optimal setting lay within the plateau region where the number of FAs identified was greatest, which closely approximated the number identified from a supervised classification (S2 Fig). Zebra herds tended to be more mobile in their half-day displacements than sable (S1 Fig), thereby supporting somewhat greater constraining distances (S2 Fig). Marginal overlap between FAs was allowed to accommodate abutting areas exploited during discrete time periods. Allowance was also made for brief excursions for travel to and from surface water, or perhaps to explore resources available elsewhere. Journeys towards water generally commenced during the early morning when they became necessary, and occasionally animals had not returned to the FA they had been exploiting by evening [11]. Hence, brief sallies beyond FA limits were labelled as temporary excursions if they endured ≤2 days.

Analyses applying the local change point (LCP) method were undertaken by means of a computer program written in TrueBASIC (http://www.truebasic.com), with the data output saved into files allowing further processing in spreadsheet software (S1 Text). Durations of settlement within FAs were assessed between the times of first entry and subsequent departure, including temporary excursions. The extent of each FA was approximated initially by ellipses excluding temporary excursions, and refined by 99% minimum convex polygons (MCP) effectively including all of the remaining locations. Exploitation intensities were derived from the ratio of the settlement time to the FA extent, excluding excursions. The proportion of days during which animals remained settled within FAs was assessed by classifying locations as falling either within FAs (excluding excursions) or during roaming sequences. The product of the exploitation intensity and proportion of days settled within FAs yielded the compound utilization intensity.

### Residence times within local circles of fixed radius

Following Barraquand and Benhamou [26], residence times (RTs) were computed for virtual circles of constant radius sliding along the sequence of foraging locations. RTs represented the summed time of all portions of the movement path located within the circles, provided that any time spent outside the circle before re-entering it was less than some threshold period. Arbitrarily, we set the radius for the circles at 1.0 km, equivalent to a patch area of 3.14 km^2^. This value is sufficient to encompass several days of foraging by the herds. The time threshold allowed out of the circle was set to 24 h to accommodate journeys to and from water completed within a day. The RT series was then segmented using the statistical procedure developed by Lavielle (2005) to identify the most likely set of change points differentiating blocks of data distinct in RT. To align the output with that obtained from the LCP method, we set the minimum duration for settlement at 5 days. To accommodate seasonal variation in movement distances, this procedure was applied to three-month bins of data, retaining a constant circle radius of 1 km within each block to allow comparison between seasons. However, to avoid arbitrary ends of the last segment of a given bin, we started the next bin at the last breakpoint date of the previous bin, therefore using natural breaks in the movement data. By using the RT as signal for the Lavielle segmentation procedure the temporal component of settlement periods was taken into account.

### Utilization intensity distributions differentiated in time

Following Benhamou and Riotte-Lambert [28], we computed the utilization intensity distribution (ID) from the RT estimated around each successive location, which corresponds with the spatial distribution of the mean RT per visit. To be consistent with the Lavielle segmentation procedure, the kernel smoothing parameter *h* was set to 333 m to obtain an effective circle radius of 1.0 km around each location. As above, we computed IDs separately for three-month bins of data, and adjusted divisions between these bins to avoid arbitrary splits of the data. To set the value of the isopleth used to define the sets of locations falling within FAs for each split of the data, we looked for an increase in slope of the total area included within putative FAs as the isopleths were expanded, indicating when outlying points or additional clusters of points became added (S3 Fig). This region was approximated using the 50% kernel. To adapt this method to FA scale and estimate settlement durations, we took into account the criteria used to delineate FAs by LCP, i.e. locations were considered to represent temporary excursions if departures from FAs persisted for less than 2 days before return to the same FA. We then re-delineated FAs using 99% MCPs as before, setting a minimum duration of 5 consecutive days for settlement. The estimation and segmentation of RT and the computation of ID were performed using the packages adehabitatLT and adehabitatHR [32] for R [30]. The extents of the FAs estimated by the 99% MCP from the LCP approach were compared with those provided by the 50% kernel from the ID method [35].

## Results

### Comparative patterns of space use

**Fig 2:**
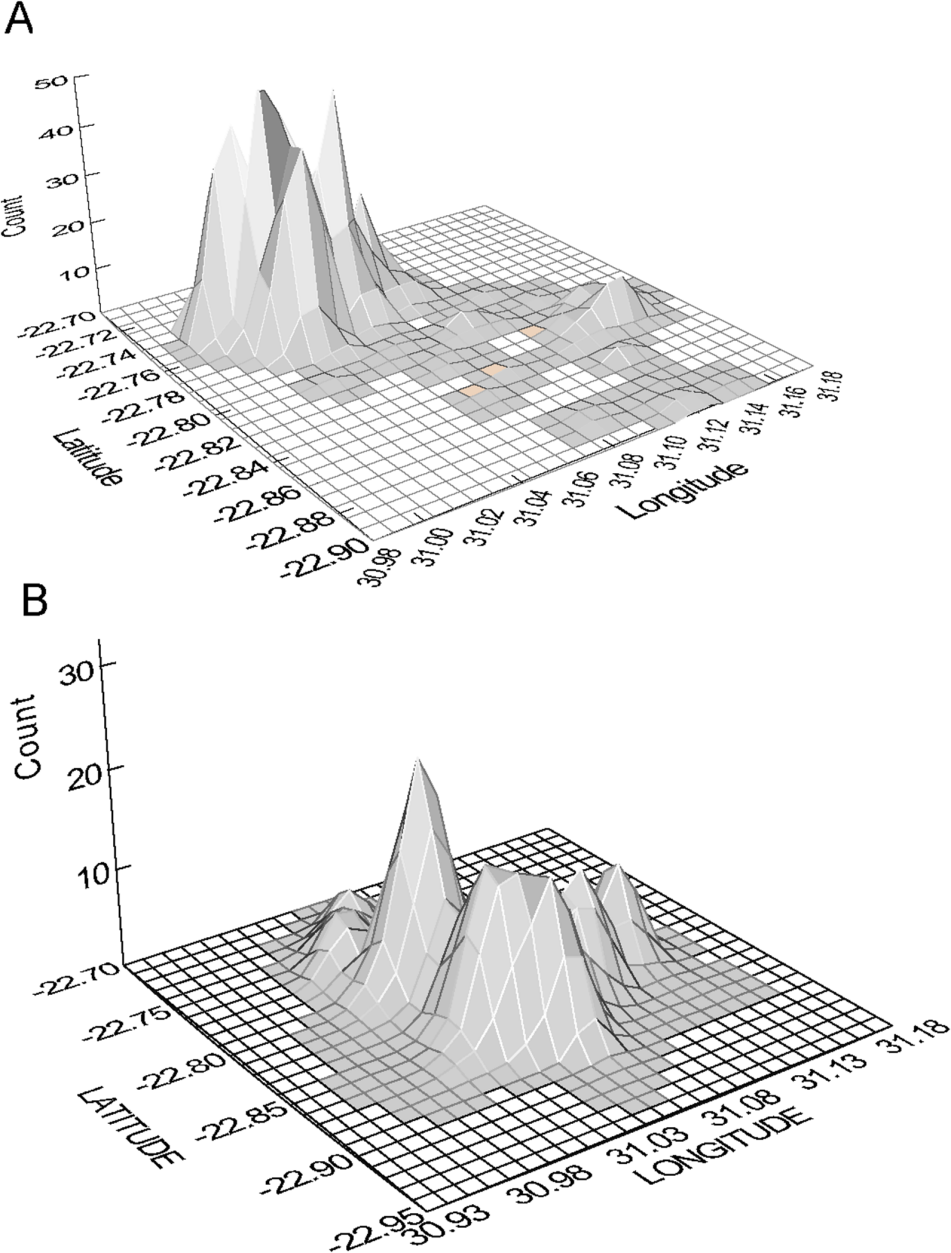
Home range utilization distributions of the northern sable antelope herd (A) and of a representative zebra herd in the same region (B), derived from locations during foraging times of day (08:00 h and 20:00 h). Note that the home range core of the sable herd is located to the north-east of the range exploited by this zebra herd.

The home range of the northern sable herd is shown plotted as a utilization distribution in Fig 2A, and compared with the home range of a zebra herd chosen for illustration because its home range overlapped with the range of this sable herd (Fig 2B). The basic home range of this sable herd, occupied for most of the year, covered an area of about 35 km^2^, with peaks of use concentrated in the north-west. During the late dry season, the sable herd moved to a separate home range 10 km away covering around 10 km^2^, which was closer to the river in the south where these animals drank every 3–4 days. Including the corridors of movement to and from the river, the total extent of the annual home range as defined by MCP covered over 150 km^2^. The home range of the representative zebra herd showed a concentration of use to the south-west of the sable home range, and encompassed a smaller total area (under 100 km^2^) because there was no seasonal shift. More generally, the spatial extents of the annual home ranges covered by the zebra herds, defined by 90% isopleths, tended to be larger than those of the sable herds (Table 2).

**Table 2:** Annual home range sizes of the seven sable and five zebra herds estimated using kernel density (90% isopleths), along with the amalgamated extent of the foraging arenas exploited by these herds over the annual cycle estimated from enclosing ellipses defined by LCP.

Species	Study area	GPS collar(s)	Annual home range (km^2^)	Amalgamated foraging arenas (km^2^)	Foraging arenas as proportion of annual range
Sable	Central	Tal01	91.3	27.8	0.304
Sable	Central	Tal02	26.2	12.4	0.473
Sable	South	PK81/140/285	38.4	29.2	0.760
Sable	South	PK144	59.6	19.6	0.329
Sable	South	PK148/284	26.4	23.2	0.879
Sable	South	PK151/286	31.7	31.5	0.994
Sable	North	PM143	163.1	52.6	0.323
Zebra	North	PM141	35.8	29.0	0.810
Zebra	North	PM142	95.0	62.0	0.653
Zebra	North	PM147	74.9	27.6	0.368
Zebra	North	PM277	345.5	99.5	0.288
Zebra	North	PM280	52.6	21.7	0.413

**Fig 3:**
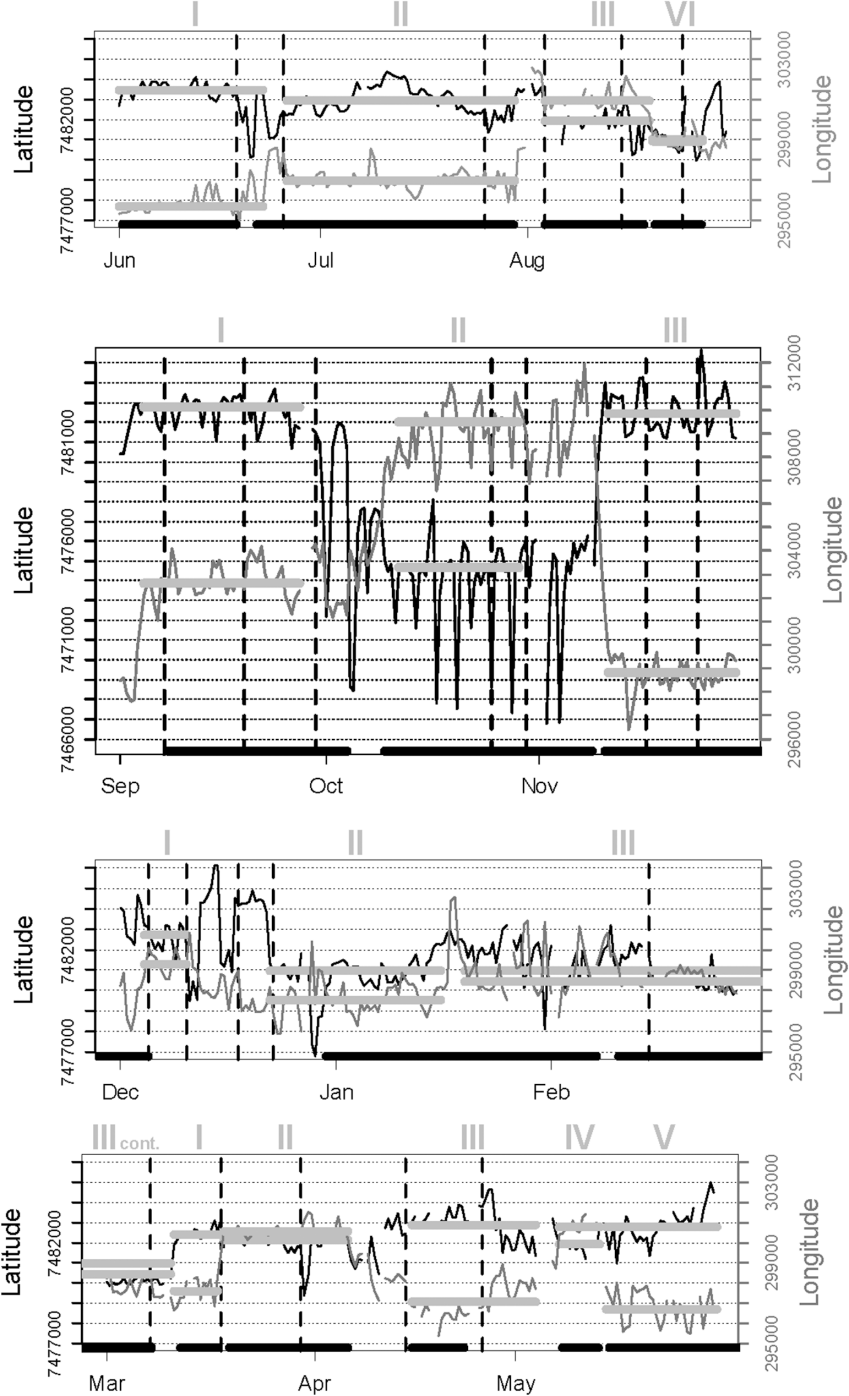
Time traces of sequential locations of the northern sable antelope herd at two foraging times per day during successive 3-month periods from June 2006 through May 2007. Grid lines are approximately 1 km apart. Wavy black line indicates the latitude trace and grey line the longitude trace. Broad grey lines indicate periods of settlement within foraging arenas for 5 days or longer indicated by local change points in distance (LCP). Broad black lines along the base indicate settlement periods obtained from the local utilization distribution of locations (ID). Vertical dashed lines indicate change points designated by the Lavielle (2005) method based on residence times around successive locations (RT);each dashed line corresponds to the beginning and end of successive segments. Latin numbers above indicate successive ordering of foraging arena occupation. Coordinates have been projected into UTM

**Fig 4:**
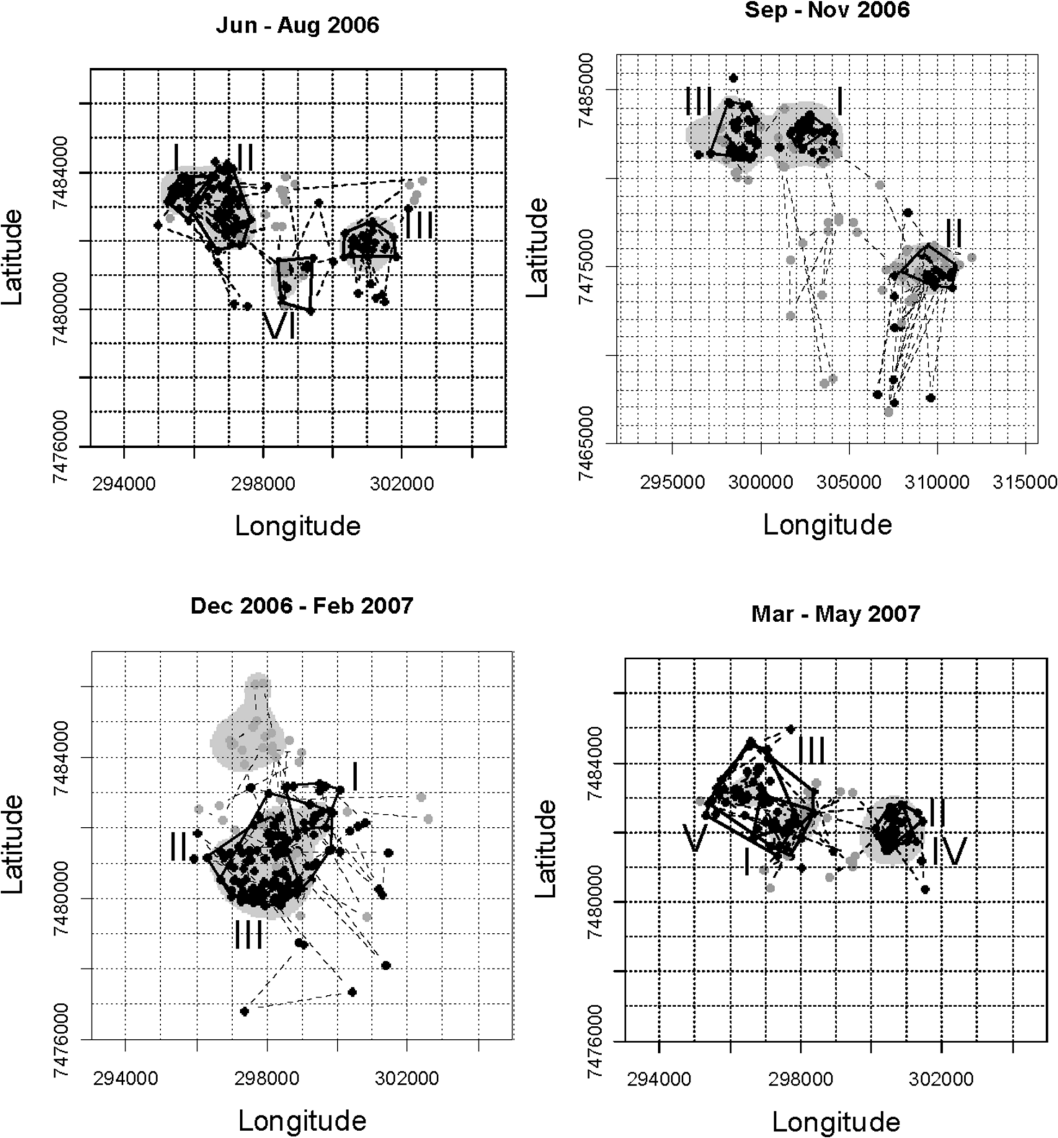
Foraging arenas occupied by the northern sable antelope herd mapped from the sequential time trace of locations from June 2006 through May 2007. Black points indicate positions assigned to periods of settlement within foraging arenas, including temporary excursions, while grey points represent roaming interludes. Black lines are minimum convex polygons delineating foraging arenas from local change points in time, while grey shadings indicates 99% kernel density estimates of the foraging arenas. In each panel the grid approximates 1 sq km. Latin numbers indicate the sequence of occupation of the foraging arenas. Coordinates are in UTM.

The FAs delineated by LCP and ID for the northern sable herd were fairly consistent, although the ID approach sometimes extended settlement periods into adjoining roaming interludes (Fig 3). Those delineated by RT applying the Lavielle method were less congruent with those demarcated by the other two methods. The RT method sometimes split settlement periods, occasionally overlooked shifts between FAs, and subdivided one roaming period. This approach failed to detect change points when the sable moved directly from one FA to the next without intervening roaming. All three methods accommodated temporary excursions for 1–2 days during settlement periods. Roaming interludes between FAs were generally brief, lasting no more than a few days, except during the late dry season and transitional months into the wet season. Despite some differences in delineation of FAs, the overall proportion of days assigned to settlement within FAs (excluding excursions) over the course of the year for the representative sable herd was closely similar from LCP (71%) and ID (68%; S1 Table). The FAs delineated by ID were closely similar to those provided by LCP, except that the bounds of the kernel density inherently extend slightly beyond the data points (Fig 4). Areas estimated from ellipses were on average 17% larger than those obtained by MCP.

**Fig 5:**
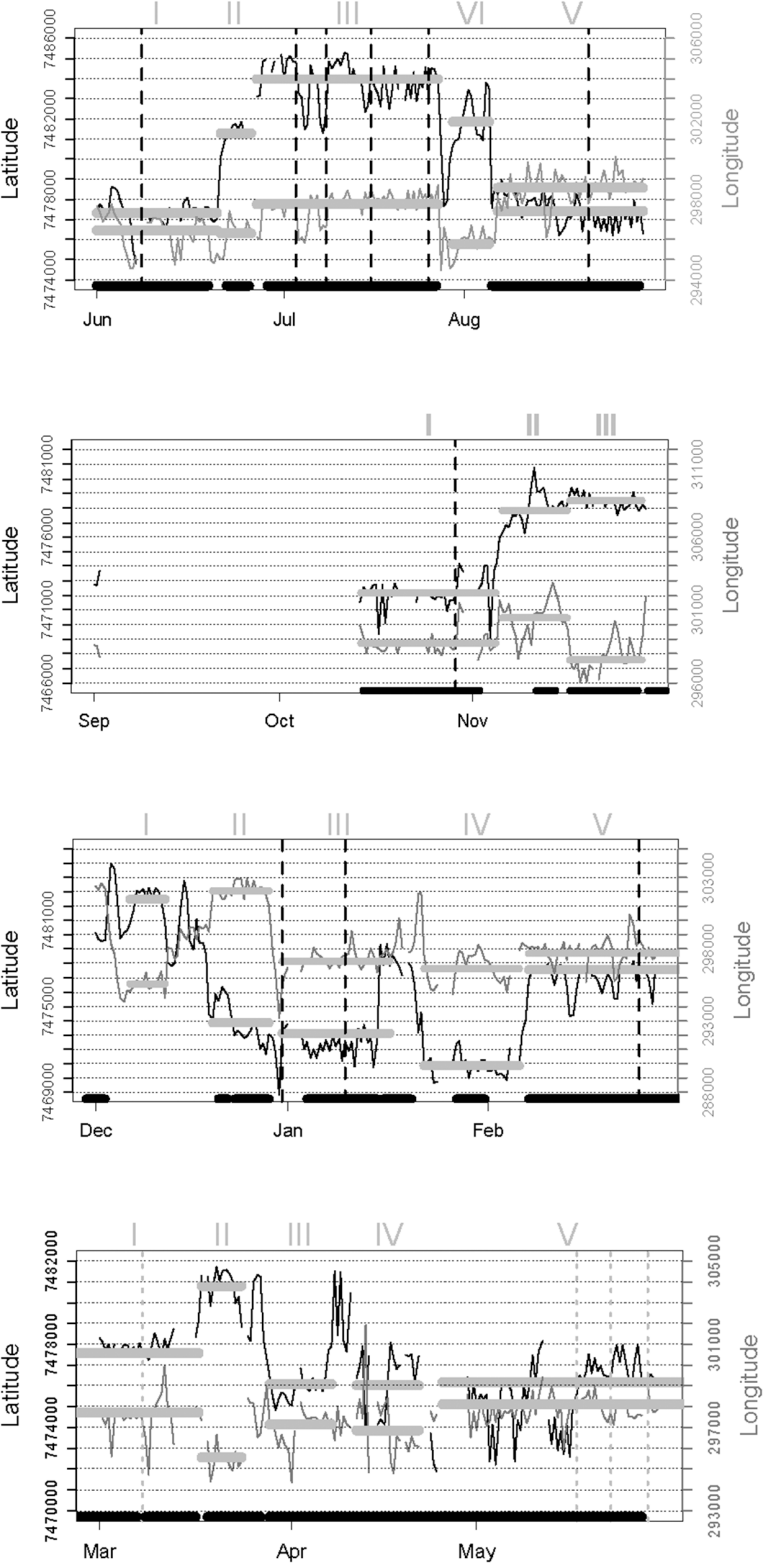
Time trace of sequential locations for a representative zebra herd spanning the same period shown for the sable herd in Fig 3; symbolic coding as in Fig 3.

**Fig 6:**
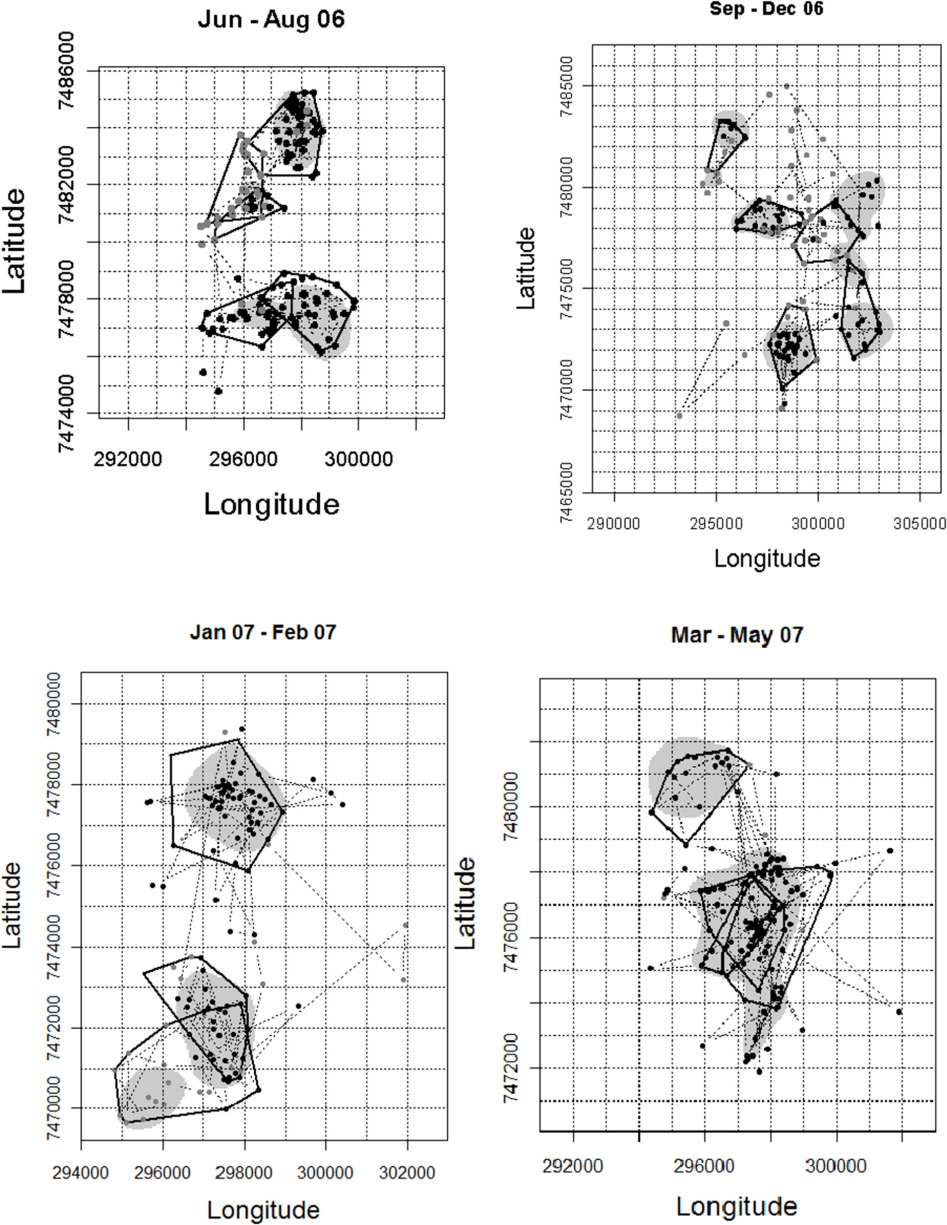
Foraging arenas occupied by the representative zebra herd derived from the time trace shown in Fig 5, with symbolic coding as in Fig 4.

When applied to GPS data for zebra herds, both the LCP approach and ID distinguished periods of settlement within FAs from interludes of roaming between them (Fig 5). Again there was quite close consistency between the settlement periods identified by LCP and ID, while RT was more deviant and tended to associate change points with brief excursions. Some distinctions in the boundaries assigned to the FAs by LCP and ID were also shown, but no real difference in spread (Fig 6)

The collared sable and zebra herds showed no consistent difference in the amalgamated extent of the foraging arenas they exploited over the annual cycle, estimated by LCP (Table 2). The annual range extent estimated by MCP also showed no consistent relationship with the extent of the FAs exploited, but rather was affected by how widely the FAs were dispersed.

### Derived metrics

**Fig 7:**
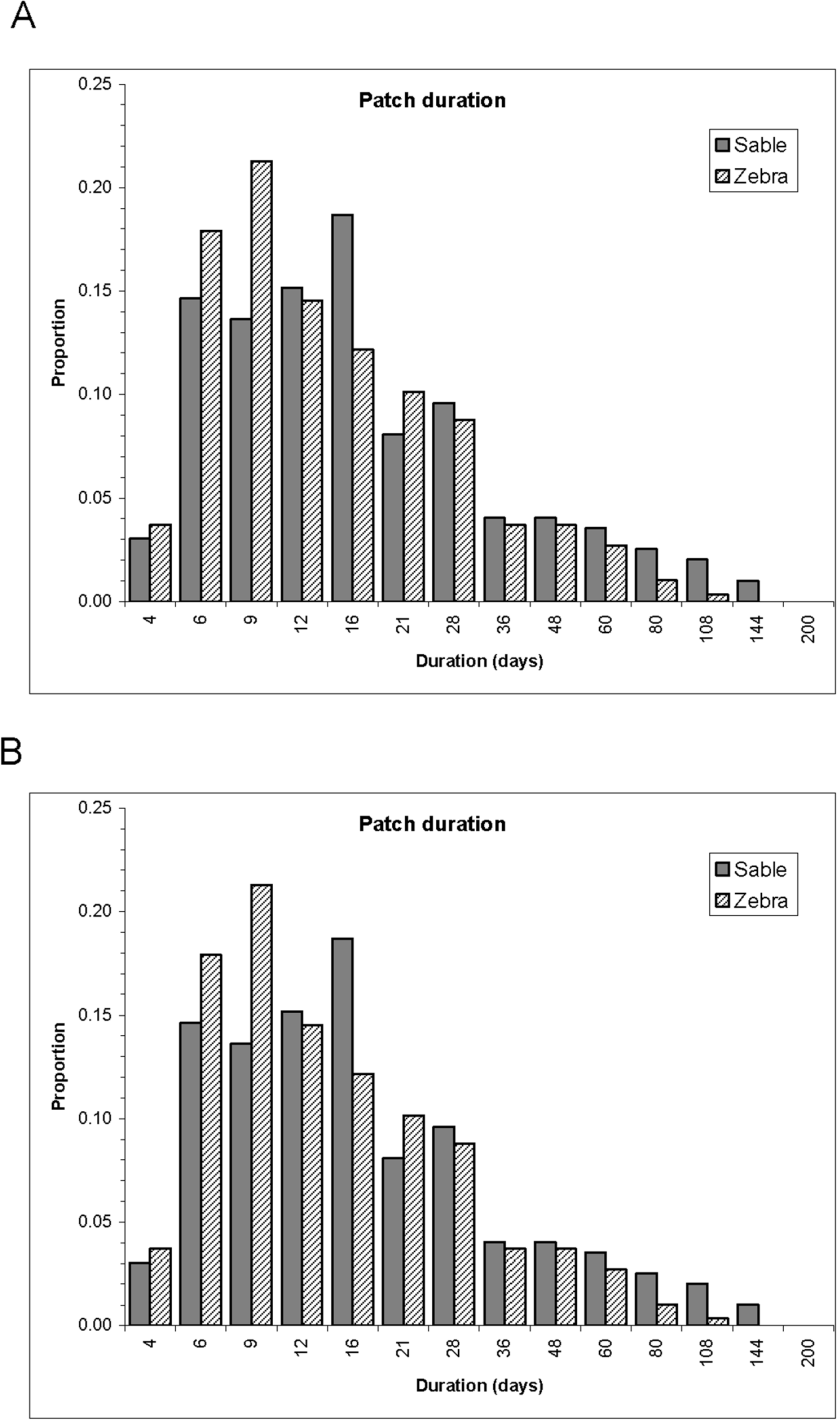
Comparison of the frequency distributions of settlement periods within foraging arenas (A) and of the extents of the foraging arenas (B) for the seven sable antelope herds and five zebra herds. Note that the temporal scale used for settlement periods is proportionately expanding.

**Fig 8:**
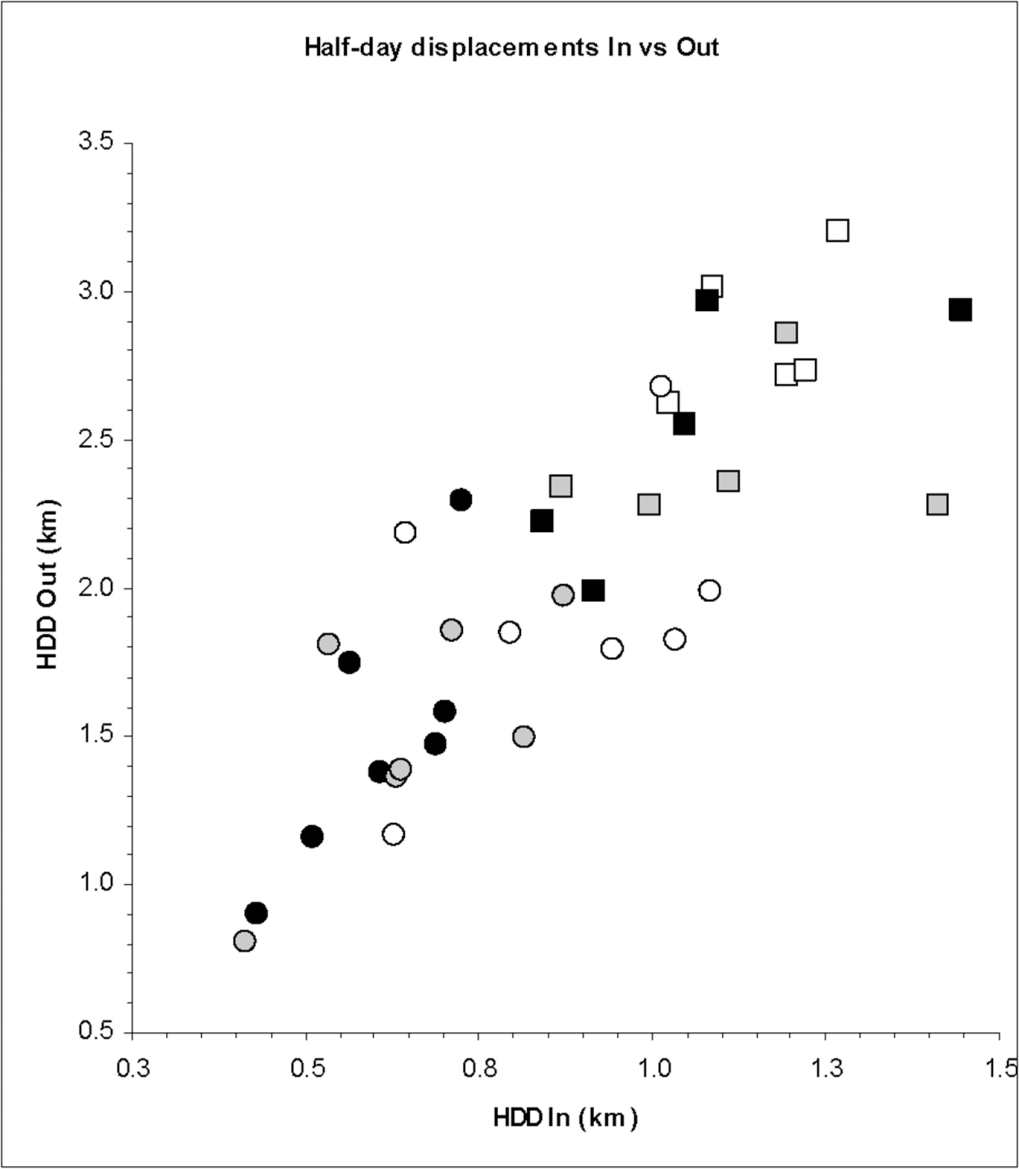
Comparison of seasonal mean values of half-day displacements while within foraging arenas with those shown during roaming interludes between foraging arenas for the seven sable antelope herds and five zebra herds. Black fill indicates wet season, grey fill early dry season, and clear fill late dry season.

For the sable herds, the range in modal durations of settlement within FAs among herds obtained from LCP, on a proportionately expanding scale, was 13–16 days, compared with 6–9 days for zebra herds (Fig 7A). Individual FAs exploited by sable herds mostly encompassed 1.5–4 km^2^, expanding to as large as 10 km^2^ during the late dry season. Zebra herds more frequently exploited FAs larger than 4 km^2^ than did sable herds, with no seasonal trend apparent. While settled within FAs, both herbivore species showed half-day displacement distances about half as great as those exhibited during roaming interludes (0.8–2.3 km vs 2–3.3 km, respectively), and zebra herds generally moved further over 12 h than did sable herds in the same season (Fig 8).

**Fig 9:**
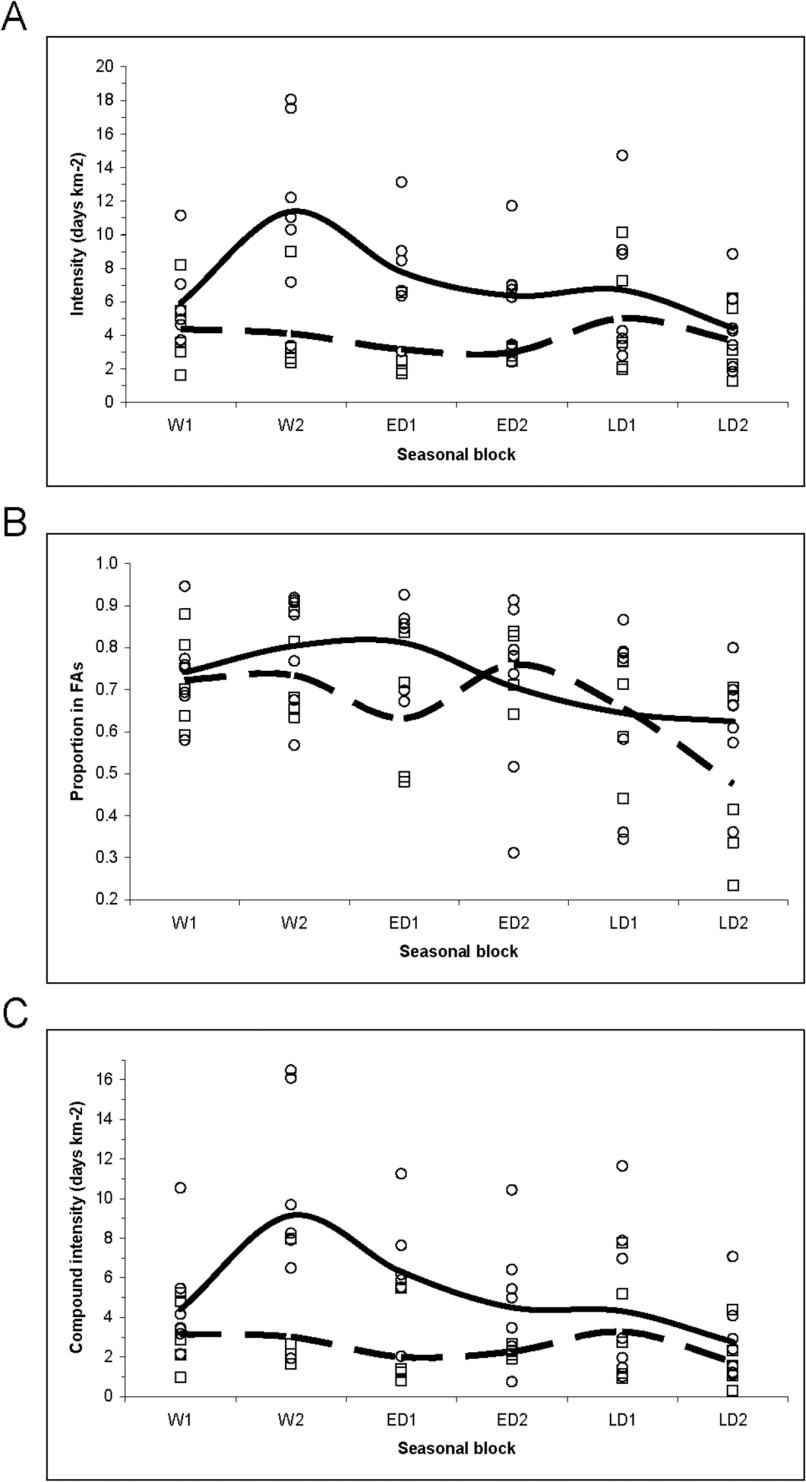
Seasonal trends in movement metrics representing the bimonthly means of the utilization intensity, in days spent per unit area, of foraging arenas (A), the proportion of days within foraging arenas compared with roaming interludes (B), and the compound intensity of use calculated as the product of days of occupation and utilization intensity (C). Circles indicate points for individual sable herds and square are points for individual zebra herds, while lines indicate mean values averaged over the sable herds (solid line) and over the zebra herds (dashed line).

Correspondingly, exploitation intensities averaged over all herds tended to be greater for sable than for zebra through all seasons, despite quite wide variation among individual herds representing these ungulate species (Fig 9A). For sable, exploitation intensities decreased from the late wet season months through the dry season. For zebra, seasonal variation was slight and two of the collared zebra herds showed substantially lowered intensities of exploitation during the early dry season months. The proportion of days spent within FAs decreased for sable through the latter part of the dry season, while for zebra a more complex pattern was shown, with lowest settled proportions during the initial months of the dry season and during the transitional months ending the late dry season (Fig 9B). The compound utilization intensity, i.e. the product of exploitation intensities within FAs and the proportion of time spent settled with FAs, showed a clear downward trend through the dry season for sable, while zebra showed little seasonal variation in this derived measure (Fig 9C). Notably, the over-riding influence came from changes in utilization intensity rather than in the settlement proportion.

## Discussion

The main contributions of this paper have been (1) to demonstrate the existence of patterns of space use associated with food benefits at a spatio-temporal scale finer than conventionally considered in home range assessment, but broader than represented by the food patches exploited during foraging spells, and (2) to present informative metrics that can be derived from patterns at this scale. Our findings challenge assumptions that the movement patterns of large mammalian herbivores can be modelled as correlated random walks, informed cognitively only by what animals have encountered recently. They support contentions that movement patterns on a day-to-day scale represent the strategic exploitation of familiar localities reliably providing suitable food, as informed by accumulated experience extending back several years [36–39]. While these beneficial places are spatially predictable, uncertainty exists in the current state of the resources presented by these localities, dependent on the erratic spatio-temporal distribution of rainfall. Sable antelope seem to favour localities that most reliably retain green foliage into the dry season, while zebra herds exploit patches that temporarily offer favourable food more opportunistically [40].

All three of the methods used delineated distinct periods of settlement extending over several days or weeks during times of the day when foraging activity predominated. However, those indicated by RT deviated somewhat from the segmentation provided by LCP and ID, seemingly influenced by the amplitude of half-day displacements as well as by the spatial consistency of sequential locations. Also, the RT approach sometimes failed to demarcate shifts between spatially separated FAs when these were made without an intervening roaming interlude. The start and end times for settlement periods within FAs provided by LCP appeared to be more consistent with our working definition of the settlement state—clusters of locations linked in time indicating that animals remained effectively stationary during successive foraging spells—than those yielded by ID. Furthermore, the change-point criterion applied in LCP is conceptually most consistent with the core concept in movement ecology, i.e. whether animals stay longer in the same place or move on elsewhere.

An inherent drawback of both RT and ID is the need to assign some arbitrary radius of the circle or distance used to assess spatial cohesion, because the spatial extents of FAs can vary quite widely, both seasonally and regionally. An advantage of LCP is that it identifies the displacements indicating departure locally, thereby allowing for temporal and spatial variation in the effective size of FAs. Although a minimal time window of five days, providing 10 locations, was adopted to assess distributions of positions around local centroids sufficiently reliably, briefer stays for periods of 3–4 days were sometimes delineated, although less reliably through being dependent on relationships with adjoining locations included within the five-day window. Constraint settings were applied to ensure that LCP did not delineate FAs too finely, due to the local stochastics of successive locations. A current disadvantage of LCP is that it has been implemented in a programming language no longer widely used. Further trials are needed to warrant translating the True BASIC program into R, which has become the computing language of choice. In the meanwhile both RT and ID are readily implementable from free software packages, and the new approach labelled T-LoCoH [41] identifying home range segments using both temporal and spatial cohesion could potentially also delineate temporary settlement periods.

Another method for identifying change points in movement behaviour is based on estimating shifts in persistence velocity and/or turning velocity [42]. To indicate settlement within a foraging arena, there would need to be a substantial and enduring change in both of these potentially inter-dependent aspects of directional persistence at an appropriate scale. In order to allow for multiple change points, this method would need to be applied within suitably restricted time windows.

The metrics of foraging performance that we derived, displayed in Figs 7–9, reflect how effectively the food benefits sought were supplied by the spatial arenas exploited. We expected that settlement durations would decrease as the amount of acceptable food diminished during the course of the dry season. In fact the settlement durations of the sable herds within foraging arenas did not change seasonally in any consistent way. This suggests that the animals widened their dietary tolerance in response to the diminishing green leaf content remaining in the herbaceous layer as grasses became dormant [40]. Correspondingly, the extent of the foraging arenas exploited by sable did tend to expand during the late dry season. As a consequence, the exploitation intensity of food resources within these FAs by sable decreased as less acceptable food remained available as the dry season advanced, and these animals correspondingly increased the time they spent roaming between FAs. We expected that zebra, as non-ruminants, would be less responsive to seasonal variation in food resources than sable, but were surprised by how little affected zebra were by the seasonal trends that occur in grass height and greenness. This reflects their dietary tolerance for amply remaining brown grass [40]. Unexpectedly, certain zebra herds spent less time settled within FAs during the initial months of the dry season rather than later in the dry season. As documented elsewhere [12], zebra also showed a marked increase in resting at the expense of mobile activities during these same months. Both patterns are intriguing, but lack any obvious explanation.

Seasonal influences on patterns of FA exploitation complement other measures of movement patterns indicating times of food stress [43]. The derived metrics also provide a foundation for addressing wider ecological issues. Spatial aspects of resource exploitation form an important aspect of niche separation among large herbivores [44]. It has been postulated that the group size of mammalian carnivores depends on the richness and dispersion of patches where prey are concentrated, according to resource dispersion hypothesis [45–46]; an objective basis for identifying the food patches herbivores exploit has now been provided. Local population densities depend on the extent of the seasonal or annual home ranges traversed (with allowance for range overlap and sharing), determined in turn by the size, spacing and intensity of exploitation of FAs within these ranges. Hence the differences in resource exploitation at foraging arena scale that we identified could help explain why sable antelope are patchily restricted in their presence within Kruger Park, compared with widely distributed zebra [47]. Accordingly, patterns at this intermediate spatiotemporal scale relating the dispersion of food resources to home range extents could help forge connections between behavioural ecology, movement ecology and population ecology.

## Supporting Information

S1 Fig Statistical distribution of half-day displacements. (DOC)

S2 Fig Establishing appropriate constraint settings for local change point delineation. (DOC)

S3 Fig Establishing appropriate isopleths for utilization intensity distribution. (DOC)

S1 Table Comparison of settlement periods and foraging arena extents for the representative sable herd obtained using alternative methods. (DOC)

S1 Text The True BASIC Program. (DOC)
