# Supplementary material for: Identifying Space Use at Foraging Arena Scale within the Home Ranges of Large Herbivores
Source: PLoS One. 2015 Jun 11;10(6):e0128821. doi: 10.1371/journal.pone.0128821 (PMC4466150; doi:10.1371/journal.pone.0128821)
Supplement: S1 Fig — (DOC) [file pone.0128821.s001.doc]

**S1** **Fig**

**Statistical distribution of half-day displacements**

Log-frequency distribution of half-day displacement distances for representative sable (A) and zebra (B) herds. Shaded region indicates segment assigned to displacements during settlement periods within foraging arenas, and open region the segment assigned to displacements during roaming interludes. On the right are the derived frequency distributions of distances from the patch centroid during periods of settlement within foraging arenas.

A

B
