# Supplementary material for: Identifying Space Use at Foraging Arena Scale within the Home Ranges of Large Herbivores
Source: PLoS One. 2015 Jun 11;10(6):e0128821. doi: 10.1371/journal.pone.0128821 (PMC4466150; doi:10.1371/journal.pone.0128821)
Supplement: S2 Fig — (DOC) [file pone.0128821.s002.doc]

**S2** **Fig**

**Establishing appropriate constraint settings for local change point delineation**

Change in the number of patches identified as foraging arenas as the constraint settings incorporated into the computer program were changed for the representative sable (A) and zebra (B) herds. Asterisks indicate the number of foraging areas distinguished from a supervised classification relative to the constraint settings that were chosen for these two herds

A

B
