# Supplementary material for: Identifying Space Use at Foraging Arena Scale within the Home Ranges of Large Herbivores
Source: PLoS One. 2015 Jun 11;10(6):e0128821. doi: 10.1371/journal.pone.0128821 (PMC4466150; doi:10.1371/journal.pone.0128821)
Supplement: S3 Fig — (DOC) [file pone.0128821.s003.doc]

**S3** **Fig**

**Establishing appropriate isopleths for utilization intensity distribution**

Increasing area covered by putative foraging arenas as the isopleth for the utilization intensity is expanded to encompass more points, showing the vague region around 50% where outlying points start becoming incorporated

|  |  |
| --- | --- |
|  |  |
