# Supplementary material for: Identifying Space Use at Foraging Arena Scale within the Home Ranges of Large Herbivores
Source: PLoS One. 2015 Jun 11;10(6):e0128821. doi: 10.1371/journal.pone.0128821 (PMC4466150; doi:10.1371/journal.pone.0128821)
Supplement: S1 Table — (DOC) [file pone.0128821.s004.doc]

**S1 Table**

**Comparison of settlement periods and foraging arena extents for the representative sable herd obtained using alternative methods**

Duration of settlement periods and corresponding foraging arena extents, comparing delineation from local change points in time (LCP) with that from the utilization intensity distribution (ID). Brackets for the last area V indicate that ID extended the settlement period beyond the end of May. Asterisks for foraging arena extents estimated by 50% kernel indicate that this method combined adjoining areas distinguished by the other two methods.

|  | Method | | Jun-Aug 2006 | | | | | Sep-Nov 2006 | | | | Dec-Feb 2007 | | | | Mar-May 2007 | | | | | Total  (days) | |
| --- | --- | --- | --- | --- | --- | --- | --- | --- | --- | --- | --- | --- | --- | --- | --- | --- | --- | --- | --- | --- | --- | --- |
| Foraging Arena |  | I | | II | III | IV | I | | II | III | I | | II | III | I | | II | III | IV | V |  |  |
| Duration (days) | LCP | 21.5 | | 34.5 | 16 | 8 | 23 | | 18 | 19 | 6.5 | | 25 | 50 | 7 | | 19 | 19 | 6.5 | 17.5 | 285 |  |
|  | ID | 17.5 | | 39 | 16 | 8 | 26 | | 31 | 25.5 | - | | 39.5 | 25 | 6 | | 19 | 9.5 | 6.5 | (17+) | 291 |  |
| Duration (excluding  excursions) (days) | LCP | 18.5 | | 32 | 13 | 7 | 18.5 | | 13.5 | 18 | 6 | | 21.5 | 46 | 7 | | 18.5 | 18.5 | 6 | 16.5 | 247 |  |
|  | ID | 17.5 | | 34.5 | 12.5 | 5 | 24.5 | | 21.5 | 24 | - | | 31 | 25 | 5.5 | | 18 | 6.5 | 5 | (16+) | 260 |  |
| Extent of MCP (km^2^) | LCP | 1.0 | | 2.7 | 1.2 | 1.2 | 2.3 | | 4.5 | 6.0 | 1.5 | | 3.5 | 6.7 | 1.2 | | 1.0 | 5.4 | 0.5 | 4.7 |  |  |
|  | ID | 1.5 | | 2.7 | 1.0 | 0.5 | 6.1 | | 5.9 | 15.1 | - | | 4.9 | 2.9 | 0.3 | | 1.0 | 0.3 | 0.5 | (4.7+) |  |  |
| Extent of 50% kernel (km^2^) | ID | - | | 4.6* | 2.1 | 0.9 | 27.7 | | 8.7 | 27.7 | - | | - | 7.7* | 1.6 | | 2.8 | 0.8 | 2.8 | - |  |  |
| Extent of ellipse (km^2^) | LCP | 1.3 | | 3.2 | 1.2 | 1.1 | 3.2 | | 5.8 | 4.1 | 1.6 | | 1.6 | 7.7 | 1.6 | | 1.3 | 7.7 | 0.7 | 6.2 |  |  |
