# Supplementary material for: Identifying Space Use at Foraging Arena Scale within the Home Ranges of Large Herbivores
Source: PLoS One. 2015 Jun 11;10(6):e0128821. doi: 10.1371/journal.pone.0128821 (PMC4466150; doi:10.1371/journal.pone.0128821)
Supplement: S1 Text — (DOC) [file pone.0128821.s005.doc]

**S1 Text**

**The True BASIC Program**

Outline of the TrueBASIC program used to identify foraging arenas from local change points in distance relative to the centroids of adjoining clusters of locations

1. READ LOCATION DATA

Data are provided in csv files containing the time sequence of decimal longitude and latitude coordinates for early morning and early evening locations, with the first entry indicating the number of records. Missing data are coded as zeros.

1. CALCULATE MEAN LONGITUDE AND LATITUDE IN 5-DAY WINDOWS POTENTIALLY INCLUDING 10 RECORDS OF EARLY MORNING AND EARLY EVENING LOCATIONS

Program skips over missing locations coded as zeros

1. IDENTIFY CANDIDATE CHANGE POINTS BY FLAGGING RECORDS IDENTIFIED AS OUTLIERS FROM PRECEDING OR SUCCEEDING CLUSTERS OF LOCATIONS

Outliers are locations further than the mean plus two standard deviations of neighbouring clusters from the centroids of these clusters, and flagged as either preceding the starting locations (S) or following the ending locations (E) of stationary periods. Allowance is made for starts or exits staggered over two time steps. Settlement locations are flagged by 0 and roaming interludes by 1.

1. ADJUST SPURIOUS STARTING POINTS OF SETTLEMENT

This establishes which of potentially multiple starting flags is retained, working forwards from the first one.

1. ESTABLISH THE CENTROID COORDINATES OF EXTENDED SETTLEMENT PERIODS

Centroid coordinates are re-calculated between the revised start and end flags

1. REASSESS DISTANCES FROM THE ADJUSTED CENTROID COORDINATES

Mean distances from the revised centroids are calculated

1. REVISE THE EXIT ASSIGNMENTS BASED ON THE ADJUSTED COORDINATES

This is done looking back up to 3 steps, and adjoining settlement blocks are joined if their centroids are less than some minimum distance apart, followed by a re-assessment of end point flags

1. REVISE THE CENTROID COORDINATES AGAIN

Centroid coordinates and related distances during settlement periods are re-calculated

1. FLAG TEMPORARY EXCURSIONS WITHIN SETTLEMENT PERIODS

Locations further than some threshold distance persisting <2 days within settlement periods are identified and flagged by 2, followed by a re-assessment of the centroids excluding these locations, revision of distances relative to the new centroids, and re-assessment of the excursion assignments

1. REVISE THE CENTROID COORDINATES, START AND END FLAGS FOR SETTLEMENT PERIODS, AND EXCURSION FLAGS

Centroid locations are re-calculated yet again and distances adjusted relative to the revised estimates, followed by adjustments to excursion assignments

1. SAVE THE OUTPUT TO A CSV FILE

The output takes the form of the longitude and latitude records, half-day displacement distances, centroid coordinates and corresponding distances derived from the initial 5-day windows, revised centroids and distances for the extended settlement periods and corresponding 0/1 assignments, followed by the finally revised estimates of these metrics.
